# Effort-Reward Imbalance and Subjective Well-Being of Chinese residency training mentors: the role of social emotions and over-commitment

**DOI:** 10.3389/fpubh.2026.1834124

**Published:** 2026-06-03

**Authors:** Huaijie Yang, Aijun Zhu, Di Xue, Jian Zheng, Jian Yang

**Affiliations:** 1 The First College of Clinical Medical Science, China Three Gorges University, Yichang, China; 2 Yichang Central People’ s Hospital, Yichang, China; 3 College of Medicine and Health Sciences, China Three Gorges University, Yichang, China; 4 Department of Cardiology, The First College of Clinical Medical Science, China Three Gorges University & Yichang Central People’s Hospital, Yichang, China

**Keywords:** burnout, Effort-Reward Imbalance, mentors, over-commitment, social emotions, standardized residency training, Subjective Well-Being

## Abstract

**Background:**

Mentors in Standardized Residency Training (SRT) experience substantial occupational stress due to demanding work environments and teaching responsibilities. This study aimed to investigate the impact of Effort-Reward Imbalance (ERI) on Mentors’ Subjective Well-Being (TSWQ), with a focus on the mediating role of Social Emotions (SES; both Positive Social Emotions [PSE] and Negative Social Emotions [NSE]) and the moderating role of Over-Commitment (OC).

**Methods:**

A cross-sectional survey was conducted with 748 Mentors. Validated scales were used to collect data on ERI, PSE, NSE, OC, and TSWQ. Parallel mediation models were employed to test mediating effects, followed by moderated parallel mediation models to examine the moderating role of OC. Regression analyses and bootstrapping methods were used to assess indirect and direct effects.

**Results:**

The parallel mediation model showed significant indirect associations of ERI with TSWQ through PSE (*β* = −0.086, *p* < 0.01) and NSE (*β* = −0.052, *p* < 0.01). The direct association between ERI and TSWQ was also significant (*β* = −0.308, *p* < 0.001). The moderated parallel mediation model revealed that OC significantly moderated the relationship between ERI and both PSE (*β* = 0.065, *p* < 0.05) and TSWQ (*β* = 0.062, *p* < 0.01), attenuating the negative associations of Effort-Reward Imbalance with these outcomes.

**Conclusion:**

The findings suggest that OC mitigates the negative impact of ERI on Mentors’ social emotions and Subjective Well-Being. Recognizing the role of OC and addressing ERI may help improve Mentors’ well-being and emotional health in SRT contexts.

## 1 Introduction

The growing global demand for high-quality healthcare has driven significant reforms in medical education (1). Standardized Residency Training (SRT), as a critical component for cultivating competent clinical physicians, has received considerable attention (2, 3). In China, the SRT system has become a fundamental cornerstone of the national healthcare strategy, aiming to comprehensively implement the core mission of fostering virtue and talent. It seeks to cultivate clinical physicians with strong professional ethics, robust clinical competence, and excellence in ideology, expertise, and work style, who are capable of independently and properly managing common and frequently occurring diseases within their specialty (4). Since its nationwide implementation in 2014, SRT has played a key role in improving clinical capabilities across various specialties and has trained a large number of healthcare professionals (1). Reports indicate that by December 2024, over the past decade, SRT in China had cumulatively trained 1.07 million residents, making a substantial contribution to the development of the national healthcare system, alleviating workforce shortages in medical institutions, reducing disparities in healthcare quality, and promoting the balanced allocation of medical talent as well as equitable development of the health sector (5). Despite the steady implementation and gradual expansion of this system, it still faces numerous challenges and areas of concern. The successful execution of SRT not only relies on residents themselves but also heavily depends on the active engagement of frontline Mentors, who typically assume dual roles as both clinical practitioners and teaching instructors. These Mentors confront considerable workloads, the demands of training quality assessment, and challenges in managing emotional well-being (6–8), with their responsibilities further complicated by additional teaching and evaluation duties.

It is evident that mentors are vital to safeguarding the integrity of the SRT system and ensuring training quality (9). In China, mentor management constitutes an important part of the SRT evaluation indicators. Every mentor who intends to supervise residents must complete pre-service mentor training at or above the hospital level and undergo hospital selection before being formally appointed. Hospitals implement dynamic management of mentors, which includes incentive and exit mechanisms (10). Mentors are required to fulfill their teaching and supervisory responsibilities for residents while simultaneously balancing their own clinical workloads, research commitments, and other teaching duties (11). Research has shown that SRT mentors commonly experience dual pressures originating from both the clinical and educational systems, including role conflict, emotional exhaustion, and a sense of imbalance and burnout arising from the insufficient recognition of their teaching efforts in career advancement (11, 12). These factors not only affect mentors’ own professional development but may also, through teaching interactions, profoundly influence the training quality and career development of residents (13).

Teacher Subjective Well-Being (TSWQ) is defined as the state of the teacher–student relationship and the teacher’s positive evaluation of their own teaching efficacy (14). Among Mentors, TSWQ is particularly salient, as these professionals must simultaneously engage in teaching, research, and the emotional labor associated with patient care (9). Although previous studies have examined the associations between residents’ Subjective Well-Being and factors such as occupational burnout and stress levels (15, 16), relatively few researchers have focused on the Mentor population in China, especially regarding the impact of Effort-Reward Imbalance (ERI) on their Subjective Well-Being.

Effort-Reward Imbalance (ERI) reflects an individual’s perception of a discrepancy between their work contributions, such as time, effort, and emotional investment, and the rewards they expect (17). The ERI model provides a well-established theoretical framework for understanding the stress experienced by professionals across various fields (18). According to this model, when individuals perceive an imbalance between effort and reward, it may lead to occupational burnout, increased turnover intention, and a decline in overall well-being. The model has been widely validated among multiple professional groups, including nurses and physicians (19–21). Given the critical role of Mentors in the SRT system, it is important to investigate their ERI status and examine how such imbalance affects their Subjective Well-Being.

Social Emotions (SES) refer to the affective states individuals experience when forming opinions and evaluating social events and situations (22). ERI in the workplace exerts a strong influence on social emotions, which are closely tied to social interactions and include both positive and negative types (23). Positive social emotions, such as compassion, gratitude, and joy, stem from fulfilling and supportive interpersonal relationships; negative social emotions, such as frustration, anger, and resentment, arise when individuals feel undervalued or unsupported (24). Research indicates that ERI adversely affects social emotions: employees coping with work stress tend to experience fewer positive emotions and more negative emotions. This imbalance can disrupt the emotional dynamics within work teams, as individuals may feel emotionally drained and develop a sense of alienation from colleagues (25). In educational and training settings, mentor support not only directly predicts trainees’ professional identity and career choice but also exerts an indirect effect through the mediating role of positive emotions (26). However, such emotional investment may also translate into emotional labor for mentors themselves. Drawing on Hochschild’s theory of emotional labor (27), mentors providing emotional scaffolding in response to the affective demands of teaching situations may need to engage in surface acting—suppressing negative emotions or exaggerating positive emotions—to maintain a supportive appearance for trainees. Empirical research has further found that mentors actively conceal their anxiety, suppress the urge to intervene, and hide emotional labor during the guidance process (28). Over time, this emotional strain may erode their empathic capacity and increase the likelihood of burnout (29).

Over-commitment (OC) refers to a psychological characteristic and behavioral tendency exhibited by individuals in responding to work demands and the rewards they receive (30). The interaction hypothesis of the ERI model conceptualizes OC as a motivational pattern characterized by excessive effort and an inability to detach from work. This pattern may increase vulnerability to adverse health outcomes resulting from high effort and low reward and may moderate the relationship between effort-reward ratio and work-related stress (31). Empirical studies indicate that increased ERI and OC are associated with higher stress and occupational burnout (32) and may reduce individuals’ life satisfaction (33). Among Mentors in China, the role of OC and its potential moderating effects on Subjective Well-Being warrant particular attention.

**Figure 1:**
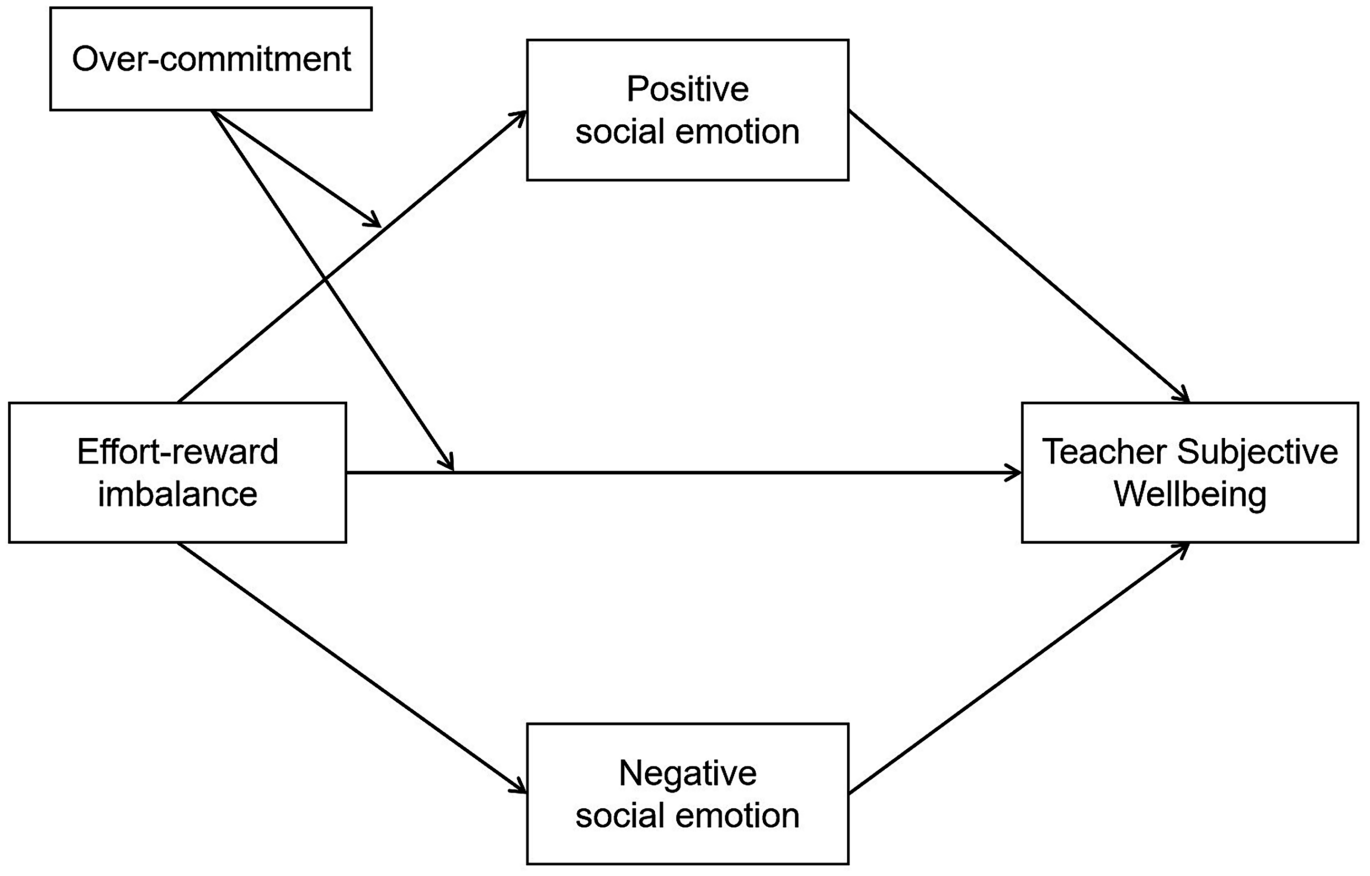
Theoretical hypothesis model.

Based on the foregoing analysis, we developed a theoretical hypothesis model (Figure 1) and proposed the following hypotheses: (1) ERI among Mentors is associated with their TSWQ; (2) SES mediate the relationship between ERI and TSWQ; (3) OC moderates the relationship between ERI and TSWQ; and (4) OC moderates the relationship between ERI and SES.

## 2 Methods

### 2.1 Participants

From October to November 2025, the research team conducted a cluster sampling survey of Mentors at two campuses of a national SRT base in Yichang, Hubei Province, using the online platform “Wenjuanxing.” The hospital is the largest medical center in the region, with over 5,300 staff members and 5,000 available beds. The inclusion criteria were as follows: (1) possession of a bachelor’s degree or higher; (2) holding a professional technical title of attending physician or above; (3) completion of hospital-organized Mentor training for residents with a passing assessment; (4) formal appointment as a Mentor by the hospital following selection by the SRT base; (5) for specialties requiring technical staff to assist in mentorship, the supervising technician must have been selected by the training base, hold an intermediate technical title for at least 3 years, and possess Mentor qualification; and (6) voluntary participation with informed consent. Exclusion criteria included: (1) clinical physicians not formally appointed as Mentors by the hospital; (2) questionnaires with missing data on key study variables, highly regular or implausible response patterns, or logical inconsistencies; (3) response time less than 300 s or greater than 1,800 s; and (4) refusal to participate in the study. The sample size was calculated using the formula: *n* = [*z*^2^ × *p* × (1−*p*)]/*d*^2^, with a 95% confidence level (*Z* = 1.96), an estimated ERI prevalence of 52.3% (19), and a permissible error of 5%, yielding a minimum sample size of 384. Considering invalid questionnaires and non-response, the sample was increased by 10%, resulting in a minimum required sample size of 423. A total of 991 questionnaires were distributed, and after excluding incomplete or invalid responses, 748 valid questionnaires were retained, corresponding to an effective response rate of 75.48%. This study was approved by the hospital Ethics Committee (Approval No. 2024-283-02).

### 2.2 Measures

#### 2.2.1 Effort-reward imbalance scale

The Chinese version of the ERI scale was used to assess the levels of effort, reward, and Over-commitment (34). The scale comprises 23 items across three dimensions: effort (6 items), reward (11 items), and OC (6 items). Responses are rated on a 5-point Likert scale (1 = strongly disagree, 5 = strongly agree), with higher scores indicating greater levels of effort, reward, or OC. The effort-reward ratio (ERR) was calculated to reflect the degree of ERI, using the formula: Total Score of the Effort Dimension/(Total Score of the Reward Dimension × 6/11), where 6/11 serves as an adjustment coefficient (the ratio of the number of items in the effort dimension to that in the reward dimension). An ERR value greater than 1.0 indicates the presence of Effort-Reward Imbalance, with higher values reflecting more pronounced imbalance. In the present study, the Cronbach’s *α* coefficients for the effort, reward, and OC dimensions were 0.867, 0.897, and 0.810, respectively.

#### 2.2.2 Social emotion scale

Social Emotions were assessed using the SES scale, which consists of two subscales: Positive Social Emotions (PSE) and Negative Social Emotions (NSE) (35). The scale contains a total of 25 items, each rated on a 5-point Likert scale (1~5), where higher values indicate greater alignment between the item content and the respondent’s experience. The PSE subscale includes 12 items across three dimensions: social pride, social gratitude, and social compassion. Higher scores indicate higher levels of positive social emotions. The Cronbach’s *α* coefficient for the PSE subscale was 0.902. The NSE subscale comprises 16 items covering four dimensions: social apathy, social complaint, social anxiety and social impetuosity. Higher scores reflect higher levels of negative social emotions. The Cronbach’s *α* coefficient for the NSE subscale was 0.871.

#### 2.2.3 Teacher Subjective Well-Being Questionnaire

Subjective Well-Being was measured using the Chinese version of the TSWQ, originally designed to assess teachers’ occupational well-being in school settings (36). The scale consists of 8 items covering two dimensions. Responses are rated on a 4-point Likert scale (1 = almost never, 4 = almost always), with higher total scores indicating higher levels of Subjective Well-Being. The TSWQ evaluates both affective and cognitive aspects of teachers’ well-being in relation to their professional roles. For the present study, the TSWQ was contextually adapted to the hospital environment for Mentors in SRT. Specifically, items referencing “school,” “students,” or “teaching situations” were modified to reflect hospital settings, residents, and clinical teaching scenarios, while maintaining the original item structure and meaning. This approach aligns with previous studies that adapted the TSWQ to different professional educational contexts. Exploratory and confirmatory factor analyses demonstrated good reliability and validity. The Cronbach’s *α* coefficient of the adapted scale was 0.918.

### 2.3 Statistical analysis

Data were analyzed using SPSS 27.0 and the PROCESS macro (Model 8). Descriptive statistics and Pearson correlation analyses were first conducted in SPSS 27.0. Simple mediation and moderated mediation analyses were performed using PROCESS 4.2. A bootstrap method with 5,000 resamples was applied to examine the mediating effects of PSE and NSE in the relationship between ERI and TSWQ using a parallel mediation model. A moderated parallel mediation model was further employed to test the moderating role of OC in the pathway from ERI to SES, as well as in the direct pathway from ERI to TSWQ. Statistical significance was determined based on a 95% bias-corrected confidence interval that did not include zero. In addition, simple slope analyses were conducted to illustrate the functional curves at different levels of OC.

## 3 Results

### 3.1 Basic information of survey participants

Baseline demographic and clinical characteristics of the study participants indicated that 53.6% were male and 46.4% were female. The majority of participants (69.1%) were aged between 36 and 49 years, while 15.5% were between 25 and 35 years. Most participants (46.9%) had 11 to 20 years of work experience, 45.9% had less than 10 years of teaching experience, and 14.3% had more than 20 years of teaching experience. Regarding educational background, 66.8% held a master’s degree, and 24.9% held a bachelor’s degree. Detailed information is presented in Table 1.

**Table 1:** Basic information of survey participants (*n* = 748).

Variables		Number	Percent (%)
Gender	Male	401	53.6
	Female	347	46.4
Age	25 ~ 35 years	116	15.5
	36 ~ 49 years	517	69.1
	50 ~ 59 years	115	15.4
Work experience	≤10 years	201	26.9
	11 ~ 20 years	351	46.9
	21 ~ 30 years	137	18.3
	≥31 years	59	7.9
Teach experience	≤9 years	343	45.9
	10 ~ 19 years	298	39.8
	≥20 years	107	14.3
Whether participating in teaching activities	No	39	5.2
	Yes	709	94.8
Professional title	Attending physician	401	53.6
	Associate chief physician	252	33.7
	Chief physician	95	12.7
Education	Bachelor’s degree	186	24.9
	Master’s degree	500	66.8
	Doctorate	62	8.3
Marital status	Married	697	93.2
	Unmarried	38	5.1
	Others	13	1.7

### 3.2 Descriptive statistics and correlation analysis of variables

As shown in Table 2, correlation analysis revealed the following relationships: PSE were significantly negatively correlated with NSE (*r* = −0.214, *p* < 0.01) and positively correlated with TSWQ (*r* = 0.462, *p* < 0.01). NSE was negatively correlated with TSWQ (*r* = −0.329, *p* < 0.01). ERI was significantly negatively correlated with PSE (*r* = −0.237, *p* < 0.01) and TSWQ (*r* = −0.443, *p* < 0.01), and positively correlated with NSE (*r* = 0.383, *p* < 0.01). OC was negatively correlated with PSE (*r* = −0.089, *p* < 0.05) and positively correlated with NSE (*r* = 0.297, *p* < 0.01), TSWQ (*r* = −0.235, *p* < 0.01), and ERI (*r* = 0.602, *p* < 0.01).

**Table 2:** Descriptive statistics and correlation analysis results for each variable (*n* = 748).

Variables	M ± SD	PSE	NSE	TSWQ	ERI
PSE	55.26 ± 6.06				
NSE	30.83 ± 8.75	−0.214**			
TSWQ	24.40 ± 4.94	0.462**	−0.329**		
ERI	0.97 ± 0.32	−0.237**	0.383**	−0.443**	
OC	19.47 ± 4.68	−0.089*	0.297**	−0.235**	0.602**

* *p* < 0.05, ** *p* < 0.01, *** *p* < 0.001, M, mean; SD, standard deviation; PSE, positive social emotions; NSE, negative social emotions; ERI, Effort-Reward Imbalance; OC, over-commitment; TSWQ, Teacher Subjective Well-Being Questionnaire.

### 3.3 Parallel mediation model

The results from Table 3 showed the testing of the parallel mediation model for the association between ERI and TSWQ through the mediating roles of PSE and NSE. The indirect association of ERI → PSE → TSWQ was found to be −0.086 (95% CI [−0.112, −0.062]), accounting for 19.28% of the total effect. The indirect association of ERI → NSE → TSWQ was −0.052 (95% CI [−0.090, −0.017]), representing 11.66% of the total effect. The direct association of ERI → TSWQ was −0.308 (95% CI [−0.369, −0.244]), accounting for 69.06% of the total effect. The total effect, combining both indirect and direct associations, was −0.446 (95% CI [−0.497, −0.387]).

**Table 3:** Testing the mediating effects of teachers’ social emotions in the Effort-Reward Imbalance and Teacher Subjective Well-Being.

Standardized effect	Influence path	Effect	Boot SE	BootLLCI	BootULCI	Relative mediating effect (%)
Indirect effect	ERI → PSE → TSWQ	−0.086	0.013	−0.112	−0.062	19.28%
	ERI → NSE → TSWQ	−0.052	0.018	−0.090	−0.017	11.66%
Direct effect	ERI → TSWQ	−0.308	0.032	−0.369	−0.244	69.06%
Total effect	Indirect effect+Direct effect	−0.446	0.028	−0.497	−0.387	100%

PSE, positive social emotions; NSE, negative social emotions; ERI, Effort-Reward Imbalance; OC, over-commitment; TSWQ, Teacher Subjective Well-Being Questionnaire. *p < 0.05, **p < 0.01, ***p < 0.001.

### 3.4 Moderated parallel mediation model

**Figure 2:**
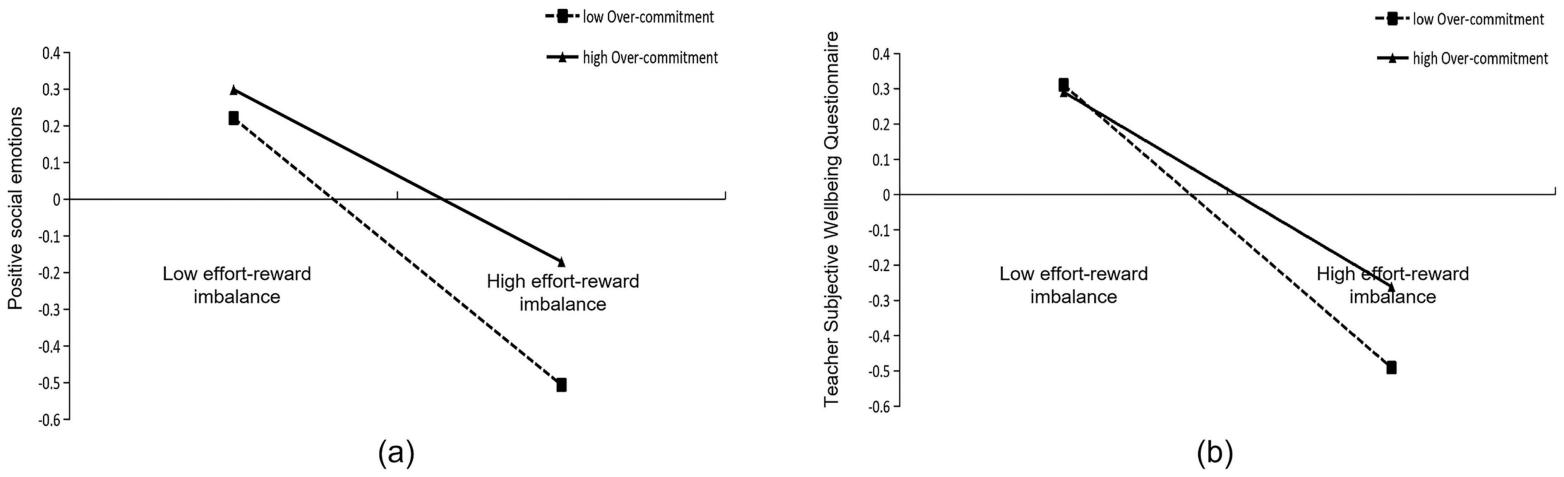
Moderating effect of over-commitment. **(a)** The moderation of over-commitment on positive social emotions. **(b)** The moderation of over-commitment on Teacher Subjective Well-Being.

The results presented in Table 4 and Figures 2a,b reveal the following findings from the moderated parallel mediation model. Analysis of PSE indicated that ERI was significantly negatively associated with PSE (*β* = −0.299, *t* = −6.707, *p* < 0.001), and OC significantly moderated this relationship (*β* = 0.104, *t* = 2.298, *p* < 0.05). The interaction term ERI × OC was significant (*β* = 0.065, *t* = 2.319, *p* < 0.05), suggesting that OC attenuates the negative association between ERI and PSE. For NSE, ERI was significantly positively associated with NSE (*β* = 0.324, *t* = 7.625, *p* < 0.001), and OC also exerted a moderating effect (*β* = 0.098, *t* = 2.281, *p* < 0.05). However, the interaction term ERI × OC was not significant (*β* = −0.022, *t* = −0.848, *p* = 0.397), indicating that OC did not significantly influence the positive relationship between ERI and NSE. Regarding TSWQ, ERI was significantly negatively associated with TSWQ (*β* = −0.338, *t* = −8.598, *p* < 0.001), PSE was positively associated (*β* = 0.352, *t* = 11.399, *p* < 0.001), and NSE had a significant negative effect (*β* = −0.136, *t* = −4.181, *p* < 0.001). OC alone was not significant (*β* = 0.052, *t* = 1.375, *p* = 0.169). The interaction term ERI × OC was significant (*β* = 0.062, *t* = 2.680, *p* < 0.01), indicating that OC significantly moderates the relationship between ERI and TSWQ, attenuating the negative association between ERI and TSWQ.

**Table 4:** Results of the moderated mediating effect.

Regression equation		Fit index			Significance of regression coefficient	
Outcome variable	Predictor variable	*R*	*R* ^2^	*F*	*β*	*t*
PSE	ERI	0.260	0.068	17.989***	−0.299	−6.707***
	OC				0.104	2.298*
	ERI × OC				0.065	2.319*
NSE	ERI	0.393	0.155	45.303***	0.324	7.625***
	OC				0.098	2.282*
	ERI × OC				−0.023	−0.848
TSWQ	ERI	0.595	0.354	81.220***	−0.338	−8.598***
	PSE				0.352	11.399***
	NSE				−0.136	−4.181***
	OC				0.052	1.375
	ERI × OC				0.063	2.680**

PSE, positive social emotions; NSE, negative social emotions; ERI, Effort-Reward Imbalance; OC, over-commitment; TSWQ, Teacher Subjective Well-Being Questionnaire. * *p* < 0.05, ** *p* < 0.01, *** *p* < 0.001.

The results presented in Table 5 illustrate the mediating role of PSE at different levels of OC. For the indirect pathway via PSE, the conditional indirect effect of ERI on TSWQ was −0.128 (95% CI [−0.181, −0.085]) at low OC, −0.105 (95% CI [−0.146, −0.072]) at mean OC, and −0.083 (95% CI [−0.121, −0.053]) at high OC. The index of moderated mediation (IMM) was 0.023 (Boot SE = 0.010, 95% CI [0.003, 0.043]). Since the confidence interval did not include zero, the indirect association via PSE was significantly moderated by OC. The positive IMM, along with the decreasing absolute value of the conditional indirect effect as OC increased, indicates that higher levels of OC attenuate the negative indirect association of ERI on TSWQ by reducing PSE.

**Table 5:** The mediating effect of positive social emotions at different levels of over-commitment.

Index	Effect	Boot SE	BootLLCI	BootULCI
eff1 (M−1SD)	−0.128	0.025	−0.181	−0.085
eff2 (M)	−0.105	0.019	−0.146	−0.072
eff3 (M + 1SD)	−0.083	0.018	−0.121	−0.053
IMM	0.023	0.010	0.003	0.043

IMM, Index of moderated mediation, OC levels: eff1 = M – 1SD, eff2 = M, eff3 = M + 1SD.

**Figure 3:**
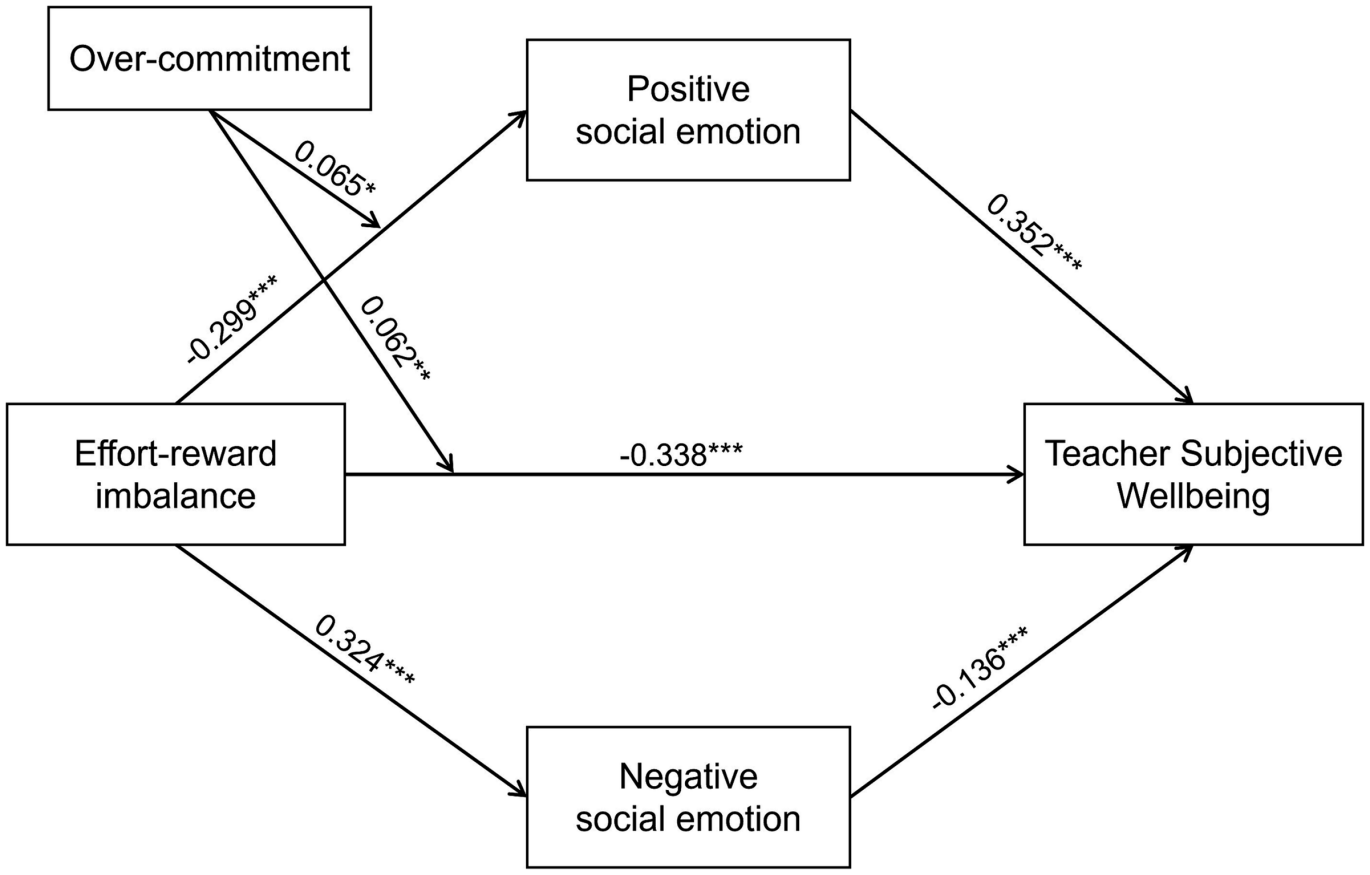
A moderated mediation model. * *p* < 0.05, ** *p* < 0.01, *** *p* < 0.001.

Figures 2a,b visually depict these relationships. Figure 2a shows that high levels of OC attenuate the negative association between high ERI and PSE, whereas low OC levels strengthen this negative relationship. Similarly, Figure 2b demonstrates that high OC weakens the negative association between high ERI and TSWQ. The final moderated model is illustrated in Figure 3. These figures highlight the pivotal role of OC in moderating the relationships between ERI and both PSE and TSWQ.

## 4 Discussion

Based on the ERI model, the present study revealed a significant association between ERI and TSWQ among Mentors in China. Incorporating SES as parallel mediators demonstrated that both PSE and NSE exert significant parallel mediating effects, indicating that ERI influences TSWQ through multiple emotional pathways. Furthermore, OC moderated the relationships between ERI and PSE, as well as between ERI and TSWQ, whereas its moderating effect on the relationship between ERI and NSE was not significant. Overall, these findings largely support the proposed moderated parallel mediation model and the corresponding research hypotheses.

### 4.1 The relationship between Effort-Reward Imbalance and Subjective Well-Being

This study highlights a critical association between ERI and TSWQ among Mentors in China. Specifically, we found that the greater the perceived imbalance between effort and reward, the lower the Mentors’ TSWQ. These findings are consistent with previous studies showing that work-related stress induced by ERI undermines the well-being of healthcare professionals (37, 38). Notably, ERI is closely related to occupational burnout (39); excessive work demands combined with insufficient recognition can lead to emotional exhaustion and reduced job satisfaction (40). Mentors are tasked not only with clinical responsibilities but also with teaching duties, balancing patient care with the management of mentor-resident relationships (9). The high-intensity work stress and emotional labor associated with these dual roles, together with ERI, significantly diminish their Subjective Well-Being. Therefore, administrative bodies should focus on improving the alignment between effort and reward for Mentors by enhancing support systems. Strategies may include refining incentive policies, clarifying the calculation of teaching workloads, implementing mentorship allowances and performance-based incentives, and strengthening professional development support in medical education. Such institutional measures can reduce the risk of ERI, prevent the marginalization of residency training responsibilities, and foster a more sustainable and supportive environment for medical education.

### 4.2 The parallel mediating role of positive and negative social emotions

The present study indicates that both PSE and NSE mediate the relationship between ERI and TSWQ. PSE, such as gratitude and pride, can buffer the negative effects of high effort and low reward among Mentors, enabling them to maintain a sense of accomplishment and well-being. In contrast, NSE, such as frustration and resentment, exacerbate the stress associated with ERI, resulting in diminished well-being. These findings align with previous research (11, 41).

According to social cognitive theory, environmental stressors do not directly influence individual outcomes but operate through internal cognitive processing, emotional appraisal, and self-regulatory mechanisms, which shape how individuals interpret experiences and subsequently regulate behavior and psychological health (42). In the current study, social emotions function as part of this self-regulatory process. Mentors perceive ERI through cognitive appraisal and internalized emotional responses. When their high-intensity work effort is inadequately rewarded, positive social emotions—such as professional accomplishment and satisfaction with interpersonal relationships—may be weakened, while negative social emotions—such as frustration and resentment—may be elicited. These emotional responses ultimately affect Mentors’ Subjective Well-Being. This interpretation is consistent with social cognitive theory. Furthermore, our findings demonstrate that PSE and NSE operate through distinct yet parallel pathways. This dual-path mechanism extends prior research on emotional labor and occupational stress in healthcare settings (43), highlighting that the depletion of PSE and the accumulation of NSE may serve as key mechanisms linking ERI to Subjective Well-Being outcomes. The findings provide empirical evidence for understanding how contextual stress in SRT can be translated into differentiated emotional experiences, shaping Mentors’ psychological well-being. Specifically, to enhance Mentors’ TSWQ, interventions can adopt a dual-path approach: on one hand, fostering a department culture and hospital environment that respect and support Mentors, strengthening PSE through organizational support and team climate; on the other hand, attending to Mentors’ mental health by reducing workload, providing psychological counseling, and promoting humanistic care to mitigate NSE.

### 4.3 The moderating role of over-commitment

Our study found that OC significantly moderates the relationship between ERI and TSWQ among Mentors. The results indicate that OC attenuates the negative association between ERI and both PSE and TSWQ. Specifically, the positive interaction coefficient suggests that for Mentors with higher levels of OC, the decline in PSE and TSWQ associated with increasing ERI is smaller than that observed in Mentors with lower OC. This finding contrasts with previous assumptions that high OC generally exacerbates work-related stress (44). A possible explanation is that highly committed individuals may possess greater resilience or receive specific support that buffers the negative effects of ERI (45); however, further research is needed to validate and explore this mechanism. By demonstrating the moderating role of OC, this study extends understanding of its interaction with work stressors. Organizational strategies should therefore address the moderating effects of OC, tailoring workload adjustments and psychological support to the psychological demands and resource needs of Mentors with varying levels of commitment and dedication. Moreover, in line with Chinese government policies on SRT, a structured mental health support system should be established for the Mentor workforce to ensure stability, high-quality development, and the enhancement of their occupational well-being.

## 5 Limitations

Although this study provides a theoretical contribution to understanding the relationships among ERI, SES, and TSWQ in the Mentor population, several limitations should be acknowledged. First, the cross-sectional study design limits causal inferences regarding the relationships among variables. Longitudinal research is needed to better understand the temporal directionality of these associations. Second, the assessment instruments primarily relied on self-report measures, which are susceptible to social desirability bias and recall bias. Future studies could incorporate objective measures of occupational burnout and well-being, as well as multi-source data such as peer evaluations and supervisor ratings. Third, the sample was limited to Mentors from two campuses of a single national SRT base, which restricts generalizability, and cluster sampling may introduce clustering effects. Future research should expand the sampling scope and implement multi-center, diverse data collection to enhance the robustness of findings. Finally, although OC was treated as a moderating variable in this study, subsequent research could explore additional factors, such as coping strategies or organizational support, to gain a more comprehensive understanding of influences on Mentors’ well-being.

## 6 Conclusion

This study applied the ERI framework to the context of postgraduate medical education in China and confirmed that ERI reduces TSWQ among Mentors, with both Positive and Negative Social Emotions serving as parallel mediators. A key novel finding is that OC, traditionally considered a risk factor, actually attenuates the negative association between ERI and PSE as well as TSWQ. This highlights the need to reconsider the role of OC in medical education contexts, suggesting that with appropriate support, OC can function as a protective rather than a risk factor. The findings provide a theoretical basis for mitigating the adverse effects of ERI on Mentors and enhancing their TSWQ, offering multiple perspectives for the ongoing optimization and development of the Mentor workforce in SRT.

## Data Availability

The datasets presented in this article are not readily available because the datasets used and/or analyzed during the current study are available from the corresponding author upon reasonable request. Requests to access the datasets should be directed to hjie-yang@ctgu.edu.cn.
